# Supplementary material for: EnzML: multi-label prediction of enzyme classes using InterPro signatures
Source: BMC Bioinformatics. 2012 Apr 25;13:61. doi: 10.1186/1471-2105-13-61 (PMC3483700; doi:10.1186/1471-2105-13-61)
Supplement: Addtional file 5 — The Java code to format the data files, evaluate and predict. The file enzml_java_code.tar.gz contains the Java code used to format database data to ARFF and XML formats, to execute cross and train-test (jackknife) evaluations and to record evaluation results to database. More information is included in the readme.txt file and the Javadoc files. The code can be used with a MySQL database. To use a different database software, other JDBC drivers might be required. [file 1471-2105-13-61-S5.gz › java_code/enzml2011/doc/uk/ac/ed/inf/enzml/test/package-tree.html]

uk.ac.ed.inf.enzml.test Class Hierarchy


---


|  |  |  |
| --- | --- | --- |
| **Overview** \| **Package** \| Class \| Use \| **Tree** \| **Deprecated** \| **Index** \| **Help** | |  |
| **PREV**   **NEXT** | **FRAMES**    **NO FRAMES**     **All Classes** |  |


---


## Hierarchy For Package uk.ac.ed.inf.enzml.test

**Package Hierarchies:**: All Packages

---

## Class Hierarchy

- java.lang.Object
  - uk.ac.ed.inf.enzml.test.**AllArffTests**- uk.ac.ed.inf.enzml.test.**AllMulanArffTests**- uk.ac.ed.inf.enzml.test.**AllMulanLearningTests**- uk.ac.ed.inf.enzml.test.**AllMulanPredictionTests**- uk.ac.ed.inf.enzml.test.**AllMulanTests**- uk.ac.ed.inf.enzml.test.**AllPreliminaryTests**- uk.ac.ed.inf.enzml.test.**AllTests**- uk.ac.ed.inf.enzml.test.**AllTrainTestsTests**- uk.ac.ed.inf.enzml.test.**AllUtilsTests**

---


|  |  |  |
| --- | --- | --- |
| **Overview** \| **Package** \| Class \| Use \| **Tree** \| **Deprecated** \| **Index** \| **Help** | |  |
| **PREV**   **NEXT** | **FRAMES**    **NO FRAMES**     **All Classes** |  |


---
